# Genetic diversity and genomic resources available for the small millet crops to accelerate a New Green Revolution

**DOI:** 10.3389/fpls.2015.00157

**Published:** 2015-03-24

**Authors:** Travis L. Goron, Manish N. Raizada

**Affiliations:** Department of Plant Agriculture, University of GuelphGuelph, ON, Canada

**Keywords:** finger millet, kodo millet, foxtail millet, barnyard millet, proso millet, little millet, New Green Revolution, biodiversity

## Abstract

Small millets are nutrient-rich food sources traditionally grown and consumed by subsistence farmers in Asia and Africa. They include finger millet (*Eleusine coracana*), foxtail millet (*Setaria italica*), kodo millet (*Paspalum scrobiculatum*), proso millet (*Panicum miliaceum*), barnyard millet (*Echinochloa* spp.), and little millet (*Panicum sumatrense*). Local farmers value the small millets for their nutritional and health benefits, tolerance to extreme stress including drought, and ability to grow under low nutrient input conditions, ideal in an era of climate change and steadily depleting natural resources. Little scientific attention has been paid to these crops, hence they have been termed “orphan cereals.” Despite this challenge, an advantageous quality of the small millets is that they continue to be grown in remote regions of the world which has preserved their biodiversity, providing breeders with unique alleles for crop improvement. The purpose of this review, first, is to highlight the diverse traits of each small millet species that are valued by farmers and consumers which hold potential for selection, improvement or mechanistic study. For each species, the germplasm, genetic and genomic resources available will then be described as potential tools to exploit this biodiversity. The review will conclude with noting current trends and gaps in the literature and make recommendations on how to better preserve and utilize diversity within these species to accelerate a New Green Revolution for subsistence farmers in Asia and Africa.

## Small millets—valuable crops neglected by the green revolution

The “Green Revolution” represents a period of massive agricultural advancement, and is often credited with saving over a billion people from starvation in the developing world (Borlaug, 2000; Evenson and Gollin, 2003). The initial focus of the Revolution was the promotion of semi-dwarf varieties of major cereal grain crops especially rice, wheat, and maize. Such modern varieties were also methodically bred to deal with environmental stresses, and in many cases produced yields several times higher than local cultivars. A highly cited example is the global success of “miracle rice” in the 1960s (De Datta et al., 1968). When faced with potential mass famine, the Punjab region of India collaborated with international advisors to introduce IR8, a semi-dwarf rice modern variety. IR8 was found to produce up to 10 times the yield of traditionally grown varieties (De Datta et al., 1968) and helped to transform India's food production from deficit to surplus; national rice production tripled accompanied by a dramatic drop in price. IR8 and its progenitors as well as other modern varieties of cereals were further exported to other regions of the world with similar results especially in Latin America and Asia (Evenson and Gollin, 2003).

However, there are regions of the world that did not experience a Green Revolution. Sub-Saharan Africa experienced a lag in the benefits of modern varieties although efforts were made for their introduction and establishment (Ejeta, 2010). Reasons for the failure are complex. Many commentators point to institutional and political difficulties that may have hindered dissemination of new technology (Ejeta, 2010). However, it is also important to consider the agroeconomic complexities of the region, where a mixture of species less common elsewhere in the world are traditionally grown (Evenson and Gollin, 2003). A wide range of climatic zones and unique farming practices with a spectrum of soil types also created a challenge. In the early part of the Green Revolution, breeding generally consisted of modifying pre-existing genetic resources of wheat, maize, and rice in which research had already been conducted by developed nations. These varieties would be further bred to incorporate additional traits to increase yields. The strategy was not applicable to many African crops where essentially no formal work existed for researchers to build upon. In fact, it has been suggested that some African farmers faced increased hardship in response to the Green Revolution as a result of a global drop in food prices caused by its massive success elsewhere (Evenson and Gollin, 2003).

**Figure 1:**
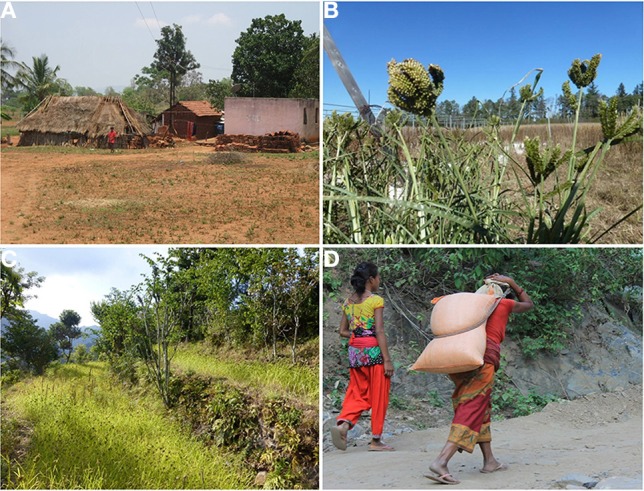
**Depictions of small millet cultivation**. **(A)** A typical subsistence small millet farm in India where the crops are grown under low input conditions and valued for their high stress tolerance. Source: M. Raizada. (**B**) Finger millet seed heads nearing maturity at the University of Guelph in Canada. The seed heads resemble the fingers of a human hand. Source: T. Goron. **(C)** Finger millet growing in a terraced field on a smallholder farm in Nepal. Source: M. Raizada. (**D**) Drudgery associated with transporting grain in the rural areas of Nepal. Source: M. Thilakarathna.

More optimistically, in the later years of the Green Revolution, research broadened to include less common food crops and began to close the gap in yield increases due to modern varieties. Locally administered organizations, such as the International Crops Research Institute for the Semi-Arid Tropics (ICRISAT), established research programs that included farmers in the dialog to strategically build a bank of genetic resources for traditionally grown species better suited to local climates and cropping systems. One group of such species is collectively known as the small millets and includes six cereal crops: finger millet (*Eleusine coracana*), foxtail millet (*Setaria italica*), kodo millet (*Paspalum scrobiculatum*), proso millet (*Panicum miliaceum*), barnyard millet (*Echinochloa* spp.), and little millet (*Panicum sumatrense*). Though all six cereals share a similar superficial classification (small grained cereals), they differ vastly in their phylogenies and continue to be grown in some of the most remote farms on Earth—thus isolation has maintained a wealth of agricultural and functional diversity. Their uses vary from animal fodder to human consumption, in which the small seeds can be ground into flour, cooked as porridge, or alternately fermented into enriched foods or alcoholic products. Where they are traditionally grown (Figure 1), small millets are highly valued for their diverse benefits and in many instances are considered nutritionally superior to other carbohydrate sources like rice and wheat (Hegde et al., 2005). Additionally, many of the small millets require very little fertilizer input as compared to more intensive grain cropping monocultures. Many reports also exist regarding their high degree of pest resistance and long-term storability, both traits which make the cultivation of small millets good insurance against famine and crop failure (Tsehaye et al., 2006; Reddy et al., 2011).

Although previously neglected, the value of small millets in modern agricultural stability has begun to be identified. Much work has been accomplished toward the development of modern varieties with the goal of better directing existing diversity toward agricultural challenges of the new millennium. The purpose of this review is to highlight the diverse traits of each crop that are valued by farmers and consumers (e.g., nutritional quality) that have potential for selection, improvement or mechanistic study, along with other phenotypes of interest, then to describe the germplasm, genetic and genomic resources available as potential tools to exploit this biodiversity. The review will conclude with noting current trends and gaps in the literature and make recommendations on how to better preserve and utilize diversity within these species to accelerate a New Green Revolution.

## Diversity of the small millets

### Finger millet (*Eleusine coracana*)

**Figure 2:**
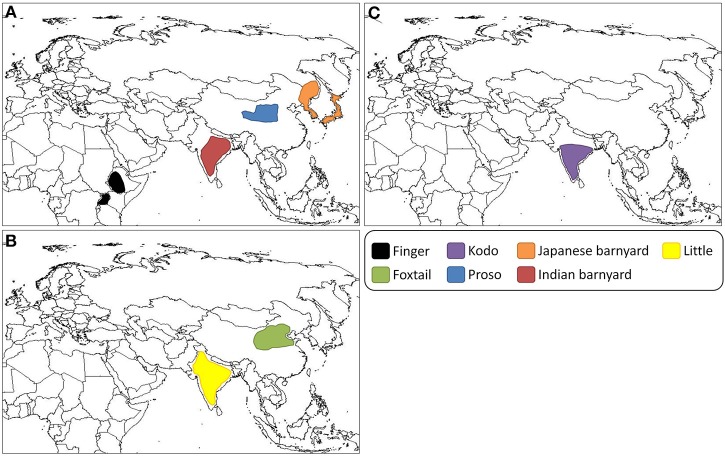
**Predicted geographic centers of domestication of the small millets**. (**A**) Finger millet is predicted to have been domesticated in Uganda and the Ethiopian Highlands (Dida et al., 2008). Proso millet was likely domesticated on the Loess Plateau, China (M'Ribu and Hilu, 1994; Hu et al., 2008, 2009). Japanese barnyard millet was likely domesticated in Japan or Eastern Asia (Yabuno, 1962). It has been suggested that Indian barnyard millet was domesticated at multiple sites across its current cultivation range in India (de Wet et al., 1983c). (**B**) Predicted sites of domestication of foxtail millet and little millet, respectively, on the North China Plain (Yang et al., 2012) and in India (de Wet et al., 1983a). (**C**) Kodo millet may have been domesticated at multiple sites across its current range of cultivation in India (de Wet et al., 1983b).

Finger millet was domesticated in western Uganda and the Ethiopian highlands (Figure 2) at least 5000 years ago before introduction to India approximately 3000 years ago (Dida et al., 2008). It is called finger millet, because the inflorescence resembles the fingers of a human hand (Figure 1). The morphology of the inflorescence can be used to differentiate between the two subspecies, *africana* and *coracana* (Dida and Devos, 2006). Each subspecies can be further divided into several races. Finger millet is an allotetraploid. Genomic donors of the “A” genome are most likely *Eleusine indica* and *Eleusine trisachya* (Liu et al., 2014). The “B” genome has yet to be uncovered, and may have been contributed by an extinct ancestor (Liu et al., 2014). It is cultivated on 1.8 million ha in India, and also fills a substantial niche in eastern Africa (Table 1) (Dida and Devos, 2006). Kenyan farmers receive a high price for the grain, often twice that of maize and sorghum (Dida and Devos, 2006). The crop is highly valued in part due to its nutritional content, being especially calcium rich. Finger millet also contains methionine and tryptophan, amino acids which are often absent in starch-based diets of some subsistence farmers (Bhatt et al., 2011). Health benefits have been investigated, including anti-cancer and anti-diabetic activity, arising, respectively, from the grain's polyphenol content (anti-oxidant activity) and high fiber (which promotes slow digestion and hence stability of blood sugar) (Chandrasekara and Shahidi, 2011a; Devi et al., 2014). The species will produce 5 tons/ha under optimum conditions (Dida and Devos, 2006) and requires very little nitrogen fertilization, with some reports indicating the most economic rate of application may be between 20 and 60 kg/ha (Hegde and Gowda, 1986; Pradhan et al., 2011). The plant is highly tolerant to drought and salt stress, though a wide diversity of stress resistance has been reported across genotypes (Uma et al., 1995; Bhatt et al., 2011). Unlike many crops consumed by subsistence farmers, finger millet has maintained high socio-economic importance in the Indian and African semi-arid tropics (Benin et al., 2004; Gull et al., 2014) and has received a level of investigation unattained by some of its cousins.

**Table 1:** **Areas where small millets are cultivated in significant quantities for human consumption**.

**Common name**	**Species name**	**Regions of cultivation**	**References**
Finger millet	*Eleusine coracana*	India, Nepal, China, Myanmar, Sri Lanka, Kenya, Uganda, Eritrea, Sudan, Zimbabwe, Zambia, Malawi, Madagascar, Rwanda, Burundi	Dwivedi et al., 2012
Foxtail millet	*Setaria italica*	China (dry northern regions), India, Nepal, Korea, Japan	Dwivedi et al., 2012
Kodo millet	*Paspalum scrobiculatum*	India	Dwivedi et al., 2012
Proso millet	*Panicum miliaceum*	India, China, Nepal, western Myanmar, Sri Lanka, Pakistan, and South East Asian countries	Hu et al., 2008; Nirmalakumari et al., 2008
Japanese barnyard millet	*Echinochloa esculenta*	Japan, Korea, Northeastern China	Yabuno, 1987
Indian barnyard millet	*Echinochloa frumentacea*	Pakistan, India, Nepal, and central Africa	Yabuno, 1987
Little millet	*Panicum sumatrense*	India, Sri Lanka, Pakistan, Myanmar, and other South East Asian countries	Hiremath et al., 1990

ICRISAT conserves 6804 finger millet germplasm accessions originating from 25 different countries. Other organizations manage germplasm banks of their own, the largest of which are summarized in Table 2. From these large collections, ICRISAT and other institutions group all genotypes according to region of origin or other parameters (Brown, 1989; Diwan et al., 1995; Hu et al., 2000; Wang et al., 2007). A subset of each group is selected that is representative of the genetic diversity of the crop: this group is termed the “core collection” and typically consists of ~10% of all available accessions. Core collections facilitate breeding by providing an efficient means to screen for desired traits from a large pool of genotypes. Mini-core collections, that represent ~1% of the total accessions, can be used by these institutions to further streamline the available genetic diversity.

**Table 2:** **Significant germplasm collections of the small millets**.

**Common name**	**Institution**	**Headquarters**	**Number of accessions**
Finger millet	• National Bureau of Plant Genetic Resources (NBPGR)	New Delhi, India	9522 (Dwivedi et al., 2012)
	• International Crops Research Institute for the Semi-Arid Tropics (ICRISAT)	Patancheru, India	6804^a^
	• All India Coordinated Minor Millet Project (AICMMP)	Bangalore, India	6257 (Dwivedi et al., 2012)
	• Kenya Agricultural Research Institute (KARI)	Muguga, Kenya	2875 (Dwivedi et al., 2012)
	• Institute of Biodiversity Conservation (IBC)	Addis Ababa, Ethiopia	2156 (Dwivedi et al., 2012)
	• USDA Agricultural Research Service (USDA-ARS)	Griffin, USA	1452^b^
	• Serere Agricultural and Animal Production Research Institute (SAARI)	Soroti, Uganda	1231 (Dwivedi et al., 2012)
	• SADC Plant Genetic Resource Centre	Lusaka, Zambia	1037 (Dwivedi et al., 2012)
	• Central Plant Breeding and Biotechnology Division, Nepal Agricultural Research Council (CPBBD)	Kathmandu, Nepal	869 (Dwivedi et al., 2012)
	• National Center for Genetic Resources Preservation	Fort Collins, USA	702 (Dwivedi et al., 2012)
	• National Institute of Agrobiological Sciences (NIAS)	Kannondai, Japan	565 (Dwivedi et al., 2012)
	• Mt. Makulu Central Research Station	Chilanga, Zambia	390 (Dwivedi et al., 2012)
	• Institute of Crop Germplasm Resources, Chinese Academy of Agricultural Sciences (ICGR-CAAS)	Beijing, China	300 (Dwivedi et al., 2012)
Foxtail millet	• Chinese National Genebank (CNGB)	Shenzhen, China	26,670 (Wang et al., 2012)
	• National Bureau of Plant Genetic Resources (NBPGR)	New Delhi, India	4330 (Dwivedi et al., 2012)
	• ORSTOM-MONTP	Montpellier, France	3500 (Dwivedi et al., 2012)
	• All India Coordinated Minor Millet Project (AICMMP)	Bangalore, India	2512 (Dwivedi et al., 2012)
	• International Crops Research Institute for the Semi-Arid Tropics (ICRISAT)	Patancheru, India	1535^c^
	• National Institute of Agrobiological Sciences (NIAS)	Kannondai, Japan	1299^d^
	• North Central Regional Plant Introduction Station, USDA-ARS	Ames, USA	1000 (Dwivedi et al., 2012)
	• Biologie Végétale Appliquée, Institut Louis Pasteur (IUT)	l'Argonne-Strasbourg, France	850 (Dwivedi et al., 2012)
	• Kenya Agricultural Research Institute (KARI)	Muguga, Kenya	772 (Dwivedi et al., 2012)
	• USDA Agricultural Research Service (USDA-ARS)	Griffin, USA	762^e^
	• Estación de Iguala, Instituto Nacional de Investigaciones Agrícolas (INIA)	Iguala, Mexico	350 (Dwivedi et al., 2012)
Kodo millet	• National Bureau of Plant Genetic Resources (NBPGR)	New Delhi, India	2170 (Dwivedi et al., 2012)
	• All India Coordinated Minor Millet Project (AICMMP)	Bangalore, India	1111 (Dwivedi et al., 2012)
	• International Crops Research Institute for the Semi-Arid Tropics (ICRISAT)	Patancheru, India	656 (Upadhyaya et al., 2014)
	• USDA Agricultural Research Service (USDA-ARS)	Griffin, USA	336^f^
Proso millet	• N.I. Vavilov All-Russian Scientific Research Institute of Plant Industry	St. Petersburg, Russian Federation	8778 (Dwivedi et al., 2012)
	• Institute of Crop Germplasm Resources, Chinese Academy of Agricultural Sciences (ICGR-CAAS)	Beijing, China	6517 (Dwivedi et al., 2012)
	• Ustymivka Experimental Station of Plant Production	S. Ustymivka, Ukraine	3976 (Dwivedi et al., 2012)
	• Yuryev Plant Production Institute UAAS	Kharkiv, Ukraine	1046 (Dwivedi et al., 2012)
	• International Crops Research Institute for the Semi-Arid Tropics (ICRISAT)	Patancheru, India	842^g^
	• Botanical Garden of the Plant Breeding and Acclimatization Institute	Bydgoszcz, Poland	721 (Dwivedi et al., 2012)
	• USDA Agricultural Research Service (USDA-ARS)	Griffin, USA	719^h^
	• North Central Reg. Plant Introd. Station, USDA-ARS	Ames, USA	713 (Dwivedi et al., 2012)
	• Estación de Iguala, Instituto Nacional de Investigaciones Agrícolas (INIA)	Iguala, Mexico	400 (Dwivedi et al., 2012)
	• National Institute of Agrobiological Sciences (NIAS)	Kannondai, Japan	302^i^
Barnyard millet (both species)	• International Crops Research Institute for the Semi-Arid Tropics (ICRISAT)	Patancheru, India	743^j^
Japanese barnyard millet	• National Institute of Agrobiological Sciences (NIAS)	Kannondai, Japan	159^k^
Indian barnyard millet	• USDA Agricultural Research Service (USDA-ARS)	Griffin, USA	232^l^
Little millet	• All India Coordinated Minor Millet Project (AICMMP)	Bangalore, India	544 (Dwivedi et al., 2012)
	• International Crops Research Institute for the Semi-Arid Tropics (ICRISAT)	Patancheru, India	466^m^
	• USDA Agricultural Research Service (USDA-ARS)	Griffin, USA	212^n^

a http://www.icrisat.org/crop-fingermillet.htm

b http://www.ars-grin.gov/npgs/index.html

c http://www.icrisat.org/crop-foxtailmillet.htm

d http://www.gene.affrc.go.jp/index_en.php

e http://www.ars-grin.gov/npgs/index.html

f http://www.ars-grin.gov/npgs/index.html

g http://www.icrisat.org/crop-prosomillet.htm

h http://www.ars-grin.gov/npgs/index.html

i http://www.gene.affrc.go.jp/index_en.php

j http://www.icrisat.org/crop-barnyardmillet.htm

k http://www.gene.affrc.go.jp/index_en.php

l http://www.ars-grin.gov/npgs/index.html

m http://www.icrisat.org/crop-littlemillet.htm

n http://www.ars-grin.gov/npgs/index.html

The morphological diversity present within finger millet is immense. For example, a range of seed colors can be produced which are correlated with protein and calcium content (Vadivoo et al., 1998). Landraces with different attributes (e.g., time to maturity, bird tolerance, drought tolerance, disease tolerance) are valued by farmers based on local agricultural complexities that reflect their productivity across multiple agroeconomic zones (Tsehaye et al., 2006). For example, in the Ethiopian highlands, three high-yield landraces were identified and further developed into the commercial lines Tadesse, Padet, and Boneya (Aduguna, 2007). During a severe drought, Tadesse finger millet was the only cereal that remained productive. Farmers received double the price for the grain as compared to maize (Aduguna, 2007). This study illustrates what can be accomplished if germplasm banks are properly utilized for the selection of desirable traits.

The degree of morphological differences in finger millet requires that even core collections to be quite large; specialized tools will be needed to simplify characterization of functional diversity. Molecular markers represent one class of such tools, including restriction fragment length polymorphisms (RFLP), amplified fragment length polymorphisms (AFLP), expressed-sequenced tags (EST), and simple sequence repeats (SSR). Very few are reported for finger millet but more are beginning to appear in the literature. Molecular markers have been utilized in attempts to characterize calcium dynamics (Yadav et al., 2014b), disease resistance (Babu et al., 2014d), and in the association mapping of various agronomic traits as well as tryptophan accumulation (Babu et al., 2014a,b). Marker-assisted research has suggested that there was little sequence diversity in finger millet populations (Muza et al., 1995; Salimath et al., 1995; Yadav et al., 2014b), but this would be surprising given the geographic diversity in which finger millet is grown. Molecular markers have enabled linkage maps of the genome to be assembled (Dida et al., 2007). While progress has recently increased, the availability of a published genomic sequence would accelerate the development of markers to assist with genotype classification and breeding. In March 2014, the Bio-resources Innovations Network for Eastern Africa Development (Bio-Innovate) announced a finger millet sequencing project (Table 3); the initial genome assembly has been completed and the full sequence is expected by the end of 2014^1^.

**Table 3:** **Small millet genomic resources and features**.

**Common name**	**Ploidy**	**Chromosome number**	**Genome size estimate (pg, 2C)**	**ESTs available from NCBI^a^**	**Genome sequence availability**
Finger millet	Tetraploid	2*n* = 4*x* = 36 (Bisht and Mukai, 2001)	3.34–3.87 (Mysore and Baird, 1997)	1982	Sequencing in progress^b^
Foxtail millet	Diploid	2*n* = 2*x* = 18 (Wanous, 1990)	1.02–1.04 (D'Ennequin et al., 1998)	66,051	Two reference genomes (Bennetzen et al., 2012; Zhang et al., 2012)
Kodo millet	Tetraploid	2*n* = 4*x* = 40 (Burton, 1940)	1.91–1.98 (Jarret et al., 1995)	29	N/A
Proso millet	Tetraploid	2*n* = 4*x* = 36 (Baltensperger, 1996)	2.08 (Kubešová et al., 2010)	211	N/A
Japanese barnyard millet	Hexaploid	2*n* = 6*x* = 36 (de Wet et al., 1983c)	N/A	0 (74 in closely-related *Echinochloa crus*-*galli*)	N/A
Indian barnyard millet	Hexaploid	2*n* = 6*x* = 36 (Wanous, 1990)	2.7 (Abrahamson et al., 1973)	0	N/A
Little millet	Tetraploid	2*n* = 4*x* = 36 (Wanous, 1990)	N/A	0	N/A

a http://www.ncbi.nlm.nih.gov/

b http://bioinnovate-africa.org/about-us/news/item/162-finger-millet-genomics-project-to-provide-researchers-with-better-tools-for-variety-production

Research illuminating the finger millet transcriptome is beginning to appear. As the crop is valued for its high calcium content, studies have characterized calcium sensing and accumulation mechanisms across genotypes differing in their grain calcium content with the use of transcriptome high-throughput sequencing (Kumar et al., 2014b; Singh et al., 2014). A similar transcriptome analysis has been conducted on salinity responsiveness (Rahman et al., 2014). To investigate mechanisms behind the crop's impressively high nitrogen utilization efficiency (NUE), the behavior of transcription factors Dof1 and Dof2 have been analyzed. It was found that in the roots of a high-protein variety, the *EcDof1*/*EcDof2* ratio was greater than that of a low protein variety, indicating a higher activation of N uptake and assimilation genes (Gupta et al., 2014a). The authors suggest that this ratio may in the future be utilized to screen other genotypes for high NUE.

Homologs of genes known to be agronomically important in major cereals, such as the transcripts described above, may assist with targeted breeding efforts in crops that are less characterized. Specifically, sequence variants of these genes may be used to develop orthologous molecular markers; those variants that correlate with desired traits may be used to screen accessions and subsequently assist in marker-assisted breeding efforts. This strategy may represent a way forward in the small millets. For example, finger millet researchers have isolated orthologs of genes known to be involved in grain amino acid composition (*Opaque 2*) and calcium content (calcium transporters, calmodulin) (Reddy et al., 2011; Nirgude et al., 2014). The researchers then associated SSR polymorphisms within these genes to characterize accessions that differed in their protein and calcium content, thus creating a targeted, cost-effective crop improvement strategy. A similar strategy to improve finger millet seed calcium content was also reported independently that focused on orthologs of calcium-binding proteins (CBPs) with extensive characterization of a seed dominant calmodulin (Kumar et al., 2014a,c). A parallel strategy has been suggested for disease resistance in finger millet based on the initial isolation of disease resistance receptors (Reddy et al., 2011; Babu et al., 2014c).

Progress has also occurred with respect to transgenic protocols for finger millet utilizing *Agrobacterium* and callus cell bombardment (Kothari et al., 2005; Ceasar and Ignacimuthu, 2009, 2011; Sharma et al., 2011; Jagga-Chugh et al., 2012; Plaza-Wüthrich and Tadele, 2012). Such techniques have allowed finger millet plants to be improved for drought and salinity tolerance (Ramegowda et al., 2012; Anjaneyulu et al., 2014; Hema et al., 2014), zinc accumulation (Cakmak, 2008; Ramegowda et al., 2013), and disease resistance (Latha et al., 2005).

### Foxtail millet (*Setaria italica*)

Named for the bushy, tail-like appearance of its immature panicles, foxtail millet has received a promising amount of research attention. Domesticated in China (Figure 2) approximately 8700 years ago, foxtail millet is considered one of the world's oldest crops and ranks second in total world millet production, providing six million tons of grain for people throughout areas in southern Europe and Asia (Li and Wu, 1996; Yang et al., 2012). It is one of the main food crops in regions of the dry north of China (Wang et al., 2012). Foxtail millet is cultivated to a limited extent in North America for silage, birdseed, and as a cover crop. It is quick to mature, able to produce seed in 75–90 days, and sometimes grown as a “catch-crop” in between the plantings of other species (Baltensperger, 2002). Herbicide-resistant lines of foxtail millet have been identified and studied in detail (Zhu et al., 2006). Additionally, the plant is quite drought resistant and tolerant to salt stress (Jayaraman et al., 2008). The cultivar “Prasad” has been identified as being particularly salt-tolerant, perhaps due to an effective antioxidant mechanism mediated by polyamine accumulation (Sudhakar et al., 2015).

As opposed to finger millet which was the result of a single domestication event (Dida et al., 2008), the history of foxtail millet is more complex. Sequence diversity of 250 Chinese genotypes was found to be quite high, averaging 20.9 alleles per locus when examined with 77 SSRs (Wang et al., 2012). Alleles clustered into two main geographic diversity centers, indicating the possibility of two domestication events within China; more work is needed to confirm this hypothesis (Wang et al., 2012). Additionally, it has been suggested that foxtail millet was independently domesticated in Europe based on archeological evidence (Jusuf and Pernes, 1985; Hunt et al., 2008; Hirano et al., 2011).

Foxtail millet is closely related to the hardy weed *Setaria viridis*, which is assumed to be its progenitor. *S. viridis*, or green foxtail, often exists in close proximity to its cultivated cousin and is problematic throughout Eurasia and North America with many reports of herbicide resistance (Morrison et al., 1989; Marles et al., 1993; Heap, 1997). Some evidence suggests genetic clustering across foxtail species is dictated primarily by region and not taxonomy, implying that interspecific hybridization between *S. viridis* and modern *S. italica* is common (Li et al., 1942; Jusuf and Pernes, 1985). Indeed, deliberate crosses between these species have resulted in resistance to a variety of herbicides (Darmency and Pernes, 1985, 1989; Wang et al., 1996; Wang and Darmency, 1997). However, agronomic traits in many of the crosses were closer to the weedy variety of *Setaria*; hybrids displayed seed shedding, spindly shoot tissue, and low yield as well as the fertility losses associated with hybridization. These reports highlight the possibility of using interspecific hybridization to study different agronomically valuable traits from wild millet relatives in a domesticated genetic background for future breeding applications.

After its domestication in China, foxtail millet spread throughout Asia, Europe, and eventually to North America (Jusuf and Pernes, 1985). Its large range has resulted in three different races, each with multiple subraces. *Moharia* is common in Europe, Russia, and the Middle East. *Maxima* can be found in Eastern China, Georgia, Japan, Korea, Nepal, northern India, and the USA where it was introduced for the purposes of animal feed. *Indica* predominates in southern India and Sri Lanka (Table 1) (Jusuf and Pernes, 1985).

An interesting feature of modern foxtail millet diversity is the global distribution of two phenotypically different varieties—the waxy and non-waxy grain type (Van et al., 2008). Waxiness in cereal grains is caused by lowered levels of amylose in the grain endosperm, which gives the grain a sticky texture when cooked (Van et al., 2008). Geographical occurrence of these two groups of foxtail millet varieties coincides with the ethnological preferences of local human populations. In East and South East Asia, some local communities are known to prefer sticky cereals (e.g., glutinous rice) driven by the use of chopsticks by these cultures—it is in these regions that the waxy millet phenotype can be found (Van et al., 2008). The non-waxy grain phenotype is more widespread, cultivated throughout Eurasia and parts of Africa (Kawase et al., 2005). Control of the phenotype is due to transposable-element (TE) insertion events interrupting amylase production, and foxtail millet has been suggested as a model for studying TE-mediated evolution (Kawase et al., 2005).

Like finger millet, there is an abundance of foxtail millet germplasm available to the scientific community (Table 2). Due to its importance in China, the Chinese National Genebank (CNGB) appears to maintain the largest collection by far, totalling 26,670 accessions as of 2012 (Wang et al., 2012). ICRISAT holds germplasm from 26 countries, and genebanks in Japan (National Institute of Agrobiological Sciences, NIAS) and the USA (USDA, Plant Genetic Resources Conservation Unit, PGRCU) ensure access to a wide range of foxtail millet diversity. Some core and mini-core collections have been assembled (Upadhyaya et al., 2008, 2011). However, considering the wide range of foxtail millet cultivation and the diversity of accessions, many more core collections should be generated, especially in China (Li et al., 1998) to facilitate breeding efforts. Diverse foxtail millet landraces may provide valuable alleles to assist in these breeding efforts. For example, landraces from the north of China are typically well-adapted to cold weather with short growing seasons, and are highly sensitive to light and temperature changes while those from southern regions grow better in high temperatures and humidity (Wang et al., 2012), demonstrating the types of useful alleles that may exist for this crop.

Foxtail millet has enjoyed more genetic characterization than the other small millets. Recently there has been a push to utilize the species as a model system for biofuel grasses. It is closely related to the bioenergy crops switchgrass (*Panicum virgatum*), napier grass (*Pennisetum purpureum*), and pearl millet (*Pennisetum glaucum*) (Doust et al., 2009). Foxtail millet has several characteristics that are valued in a model system—a small genome (~490 Mbp), small plant size, and a quick generation time, unusual for C4 grasses. As a result, two full reference sequences have been compiled using genotypes Yugu1 and Zhang Gu (Bennetzen et al., 2012; Zhang et al., 2012). In these studies, the authors also created high-density linkage maps with another foxtail millet line and green foxtail, and examined the evolution and mechanisms of C4 photosynthesis in detail (Bennetzen et al., 2012; Zhang et al., 2012).

Instigated by the newly available sequence data, research in foxtail millet molecular genomics continues to rapidly progress. Many genetic markers have been reported and utilized in foxtail millet to generate maps, analyze DNA polymorphisms, evolutionary origin(s), and relatedness to other cereals for future crop improvement efforts (Wang et al., 1998; Schontz and Rether, 1999; Jia et al., 2009; Yadav et al., 2014a). A large library of markers consisting of intron-length polymorphisms (ILPs) has been generated, in part enabled by an abundance of EST data which can be used to generate flanking primers (Muthamilarasan et al., 2014). Initial work toward marker-based, high-throughput genotype identification has been accomplished (Gupta et al., 2012; Pandey et al., 2013). For example, an allele-specific single nucleotide polymorphism (SNP) coding for a dehydration responsive element binding (DREB) gene was shown to associate with stress tolerance (Lata et al., 2011). The SNP has potential in marker-assisted breeding selection, and was validated in a foxtail millet core collection in which the allele was found to account for 27% of total variation of stress-induced lipid peroxidation (Lata and Prasad, 2013). In an association mapping study, eight SSR markers were found to correlate with nine different agronomic traits (Gupta et al., 2014b). ESTs and peptides have been identified which are differentially expressed between salt tolerant and non-tolerant cultivars (Veeranagamallaiah et al., 2008; Puranik et al., 2011). A genome-wide transcriptome has been generated after exposure to drought stress, in which regulatory roles of small interfering RNAs and non-coding RNAs were described (Qi et al., 2013). From this study, 2824 annotated genes were identified with drought-responsive expression patterns. Such comprehensive studies should be extended to other stress pathways for better characterization of available foxtail millet germplasm. The data might also be used to design useful millet microarrays. Using the reference genomes described above, research groups have begun to re-sequence genotypes of foxtail millet and identify vast libraries of SNPs and other markers (Bai et al., 2013; Jia et al., 2013). This information has been used to classify landraces according to flowering time, yield attributes, *waxy* character, and other agronomically important traits (Jia et al., 2013; Bai et al., 2013). The re-sequencing of diverse foxtail millet germplasm should continue as a strategy to aid marker-assisted breeding efforts. Much work has also been accomplished in the behavior of transcription factors in foxtail millet under a variety of stressful conditions, details of which have been conveniently compiled in the database “FmTFDb” (Bonthala et al., 2014). The availability of this data is expected to greatly accelerate functional genomics in all small millet species.

Lastly, transgenic protocols have been developed for foxtail millet, with both *Agrobacterium* (Wang et al., 2011) and callus bombardment methods reported (Kothari et al., 2005; Ceasar and Ignacimuthu, 2009; Plaza-Wüthrich and Tadele, 2012), enabling some potentially useful molecular analyses. In one study, a pollen-specific gene has been altered to impair anther function by a co-suppression mechanism (Qin et al., 2008) which might be adapted for the development of male-sterile plants, valuable in breeding foxtail millet hybrid varieties.

### Kodo millet (*Paspalum scrobiculatum*)

Kodo millet was domesticated roughly 3000 years ago in India (Figure 2), the only country today where it is harvested as a grain in significant quantities, mainly on the Deccan plateau (Table 1) (de Wet et al., 1983b). The grain contains a diverse range of high-quality protein (Geervani and Eggum, 1989; Kulkarni and Naik, 2000), and has high anti-oxidant activity (anti-cancer) even when compared to other millets (Hegde and Chandra, 2005; Hegde et al., 2005; Chandrasekara and Shahidi, 2011b). Like finger millet, kodo is rich in fiber and hence may be useful for diabetics (Geervani and Eggum, 1989). It is drought tolerant and can be grown in a variety of poor soil types from gravelly to clay (de Wet et al., 1983b; M'Ribu and Hilu, 1996). Most genotypes take 4 months to mature (de Wet et al., 1983b). Like foxtail millet, a weedy counterpart of kodo exists and is problematic throughout old-world farming systems especially in damp areas (de Wet et al., 1983b; Becker and Johnson, 2001). It is believed that kodo was probably first harvested as a weed alongside other cereals like rice, perhaps leading to multiple domestication events of the millet across its current range (de Wet et al., 1983b). This practice continues in parts of Africa where the weed is also sometimes harvested during famine (de Wet et al., 1983b; Neumann et al., 1996; Ogie-Odia et al., 2010). In Africa, kodo is referred to as black rice or bird's grass (M'Ribu and Hilu, 1996). Limited molecular marker analysis has shown that kodo millet genotypes cluster by African vs. Indian origin (M'Ribu and Hilu, 1996).

Kodo millet is divided into three races (*regularis, irregularis*, and *variabilis)* based on panicle morphology (de Wet et al., 1983b). In southern India, there are small (*karu varagu*) and large seeded (*peru varagu*) varieties recognized, often grown together in the same field (de Wet et al., 1983b). General morphological variability is high, with large variance reported in many phenotypic parameters such as time before flowering, tiller number, and yield (Subramanian et al., 2010; Upadhyaya et al., 2014).

Kodo millet is a crop that might be described as incompletely domesticated, with some authors calling the cereal “pseudo-cultivated” (de Wet, 1992; Blench, 1997). As such, systematic breeding of kodo millet remains neglected but limited efforts have shown promise. Various metrics of plant productivity including dry fodder yield, plant height, and grain yield have revealed good heritability; improvement of these traits has been observed through breeding, with four highly productive genotypes thus far identified (Upadhyaya et al., 2014). Pathogen resistance has been noted as a good breeding target, in particular resistance to smut (*Sorosporium paspali* and *Ustilago* spp.) and rust (*Puccinia substriata* Ellis and Barth), which are both major hindrances of kodo yield (Upadhyaya et al., 2014). Another potential target for breeding may be resistance to the fungi *Aspergillus flavus* and *Aspergillus tamari* which produce cyclopiazonic acid that can cause sleepiness, tremors, and giddiness in those that consume infected grain, known as “kodua poisoning” (Rao and Husain, 1985). Grain lodging can occur before harvest, therefore an earlier maturity time might also be targeted (de Wet et al., 1983b). It is also interesting that some cultivated landraces have maintained the perennial nature of their wild ancestor and continue to initiate culms following the maturity of older shoots (de Wet et al., 1983b). If this regeneration trait can be encouraged through breeding and hybridization, it may reduce fertilization inputs and labor.

Unfortunately, no genetic or molecular maps of the kodo millet genome appear to be available (Dwivedi et al., 2012), likely because of the problem of persistent cross-hybridization with its wild relatives. Molecular markers for kodo millet are few, but have been utilized in characterizing diversity and phylogeny (M'Ribu and Hilu, 1996; Kushwaha et al., 2015). There has been some preliminary work in miRNA target site prediction using ESTs from kodo (Babu et al., 2013). In this study, target genes were found be involved in carbohydrate metabolism, cellular transport, and as structural proteins, but a severe lack of kodo DNA information limited this study; the closely-related rice genomic sequence was used for binding-site prediction. With respect to transgene methodology for kodo, the media conditions for callus regeneration protocols have been investigated; regenerated plantlets were successfully grown to maturity in soil (Ceasar and Ignacimuthu, 2010).

ICRISAT conserves 656 accessions of kodo millet, and a core collection has been established that reflects the phenotypic diversity of the entire collection (Upadhyaya et al., 2014). Some universities also maintain large kodo millet seed banks, a good example being the University of Agricultural Sciences in Bangalore (Ceasar and Ignacimuthu, 2010). As the crop is not significant outside of India, there are few reports of other banks with substantial numbers of accessions (Table 2). However, some organizations do keep collections for the purposes of studying the species as a weed as noted above; the US Department of Agriculture has 336 accessions in their National Plant Germplasm System (GRIN)^2^. While seed of African origin does exist in some of these sources, it is rare. Better coverage and ecological exploration of the African continent would help to reveal and preserve diversity of valuable traits which might otherwise be missed by international scientists.

### Proso millet (*Panicum miliaceum*)

Proso millet, also called broomcorn and common millet, was domesticated in Neolithic China as early as 10,000 years ago (Figure 2) (Lu et al., 2009). The sequence diversity within proso provides evidence for a single site of domestication in the Chinese Loess Plateau (M'Ribu and Hilu, 1994; Hu et al., 2008, 2009). Proso millet expanded across Eurasia and was introduced to North America in the 1700s where it is now primarily used for animal fodder and birdseed (Bagdi et al., 2011). Proso is the true millet referenced in classical European and Middle Eastern sources, referred to by ancient Romans as “*milium*” (Smith, 1977). Archeological evidence of proso in Eastern Europe dating to 8000 years ago raises the possibility of a secondary independent domestication event, but additional study is needed to confirm this observation (Hunt et al., 2008, 2011). Proso millet was important in the diets of humans across Eurasia prior to the introduction of wheat, barley and potatoes (Kalinova and Moudry, 2006). Today it is only consumed in significant quantities in India (where it is known as *pani varagu* in Tamil), Nepal, western Myanmar, Sri Lanka, Pakistan, and South East Asian countries (Nirmalakumari et al., 2008). A weedy variety is widespread, which is likely the result of field escape and not due to the spread of the wild ancestor (McCanny and Cavers, 1988). Recent molecular analysis using chromosomal *in situ* hybridization has implicated *Panicum capillare* or a close relative as one of the genetic ancestors of proso (Hunt et al., 2014).

The benefits of consuming proso include its high protein content which ranges from 11.3 to 17% of grain dry matter (Kalinova and Moudry, 2006). Genotypic diversity in protein content and amino acid profile has been observed (Kalinova and Moudry, 2006). Like other small millets, the applicability of the grain in preventing cancer, heart disease, and managing liver disease and diabetes has been investigated with promising results (Nishizawa and Fudamoto, 1995; Nishizawa et al., 2002; Park et al., 2014; Zhang et al., 2014). There may be additional untapped phytochemical value as indicated by a wide range of genotype-specific grain colors (Zhang et al., 2014).

Proso millet is well-adapted to dry sandy soils, and might be the earliest dryland-farming crop in East Asia (Baltensperger, 2002; Lu et al., 2009). It may have the lowest water requirement of any cereal, able to produce harvestable grain with only 330–350 mm of annual rainfall (Baltensperger, 2002; Seghatoleslami et al., 2008; Hunt et al., 2011). Proso millet matures quickly within 60–90 days, a feature that contributes to its drought resistance and also makes it a good catch-crop (Baltensperger, 2002; Hunt et al., 2014). Genotype has been shown to affect drought tolerance by influencing harvest-index, yield, and water use efficiency (WUE) (Seghatoleslami et al., 2008). In the latter study, a hybrid genotype outperformed local varieties, validating the potential in breeding highly WUE proso millet. Preliminary work in characterizing proso miRNAs has been accomplished with the goal of understanding mechanisms responsible for the cereal's impressive drought resistance (Wu et al., 2012). Despite its drought tolerance, proso is best adapted to temperate latitudes unlike other small millets. It grows further north than any other millet up to a latitude of 54°N, and at elevations as high as 3500 m (Baltensperger, 2002). Substantial salinity tolerance has been reported in proso but with significant varietal diversity, with some especially tolerant varieties reported (Sabir et al., 2011; Liu et al., 2015). A higher sodium concentration in roots compared to shoots has been suggested as a biomarker for future breeding efforts (Sabir et al., 2011; Liu et al., 2015).

Cultivated proso millet is divided into five races (Reddy et al., 2007). Race *miliaceum* resembles wild proso with large, open inflorescences and sub-erect branches with few subdivisions. *Patentissimum* is very similar to *miliaceum* with narrow, diffuse panicle branches. These two races are found across the entire Eurasian range of proso, and are considered primitive. *Contractum, compactum*, and *ovatum* have more compact inflorescences which are drooped, cylindrical, and curved, respectively (Reddy et al., 2007). ICRISAT holds 842 accessions from all five races (Table 2) (Reddy et al., 2007). The diversity of this collection has been characterized in terms of flowering time, plant height, panicle exsertion, and inflorescence length (Reddy et al., 2007). Other significant collections of proso are summarized in Table 2. Perhaps the largest collection of proso is held by the N.I. Vavilov All-Russian Scientific Research Institute of Plant Industry in St. Petersburg, with roughly 8778 accessions as of 2012 (Dwivedi et al., 2012). Aside from ICRISAT (Upadhyaya et al., 2014), few proso millet core collections appear to exist for breeding purposes. Preliminary diversity clustering based on agronomic traits was performed on the Chinese collection for the purpose of SSR-based characterization (Hu et al., 2009). Perhaps the Chinese subset of 118 landraces could be repurposed and slightly modified to become a true core collection. Explant regeneration techniques have been published for proso, allowing transgenic work to be explored in the future (Plaza-Wüthrich and Tadele, 2012).

The genetic sequence diversity of proso has been examined to a limited degree. The sequence diversity is moderate to high (Karam et al., 2006; Cho et al., 2010; Hunt et al., 2011), perhaps due to continuing hybridization with wild varieties (Colosi and Schaal, 1997). Molecular markers in proso have often been derived from the available sequence data of related species including switchgrass, rice, wheat, barley and oat (Hu et al., 2009; Rajput et al., 2014). AFLP markers have shown promise in grouping proso based on biotype, but were insufficient in differentiating between wild and cultivated varieties (Karam et al., 2004). To the best of our knowledge, no genetic or molecular maps of the proso millet genome are available (Dwivedi et al., 2012).

Like kodo millet, waxy varieties of proso grain exist and are preferred in some areas of Asia because of their glutinous nature—again to facilitate consumption with chopsticks (Graybosch and Baltensperger, 2009). Clustering by geographical sequence diversity corresponds with this regional preference (Hu et al., 2008). Like other glutinous cereals, waxy types of proso have no detectable amylose in the seed endosperm, due to a mutation in the *Waxy* gene (Hunt et al., 2010). Molecular markers have been developed to identify these waxy genotypes and breed glutinous varieties that are highly valued by consumers (Araki et al., 2012). Proso has been compared to maize in its ethanol production ability, and fermentation efficiency was found to be the highest in waxy varieties (Rose and Santra, 2013). The authors suggest that encouraging the fermentation of proso millet could help stabilize its price in the USA where it is already grown for birdseed and fodder. Finally, proso millet has been utilized as a model organism for C4 carbon metabolism, specifically in the study of aspartate aminotransferase and malate translocation which both contribute to the higher efficiency of C4 photosynthesis (Taniguchi et al., 1995; Taniguchi and Sugiyama, 1996, 1997; Sentoku et al., 2000).

### Barnyard millet (*Echinochloa* spp.)

Although sometimes referred to as a single taxonomic group, barnyard millet is composed of two separate species belonging to the genus *Echinochloa*. *Echinochloa esculenta* (syn. *Echinochloa utilis, Echinochloa crusgalli*) is cultivated in Japan, Korea, and the northeastern part of China while *Echinochloa frumentacea* (syn. *Echinochloa colona*) is found in Pakistan, India, Nepal, and central Africa (Table 1) (Yabuno, 1987; Wanous, 1990). Both species have overlapping morphological traits that make differentiation problematic. Visual identification is only possible based on the presence or absence of an awn and subtle differences in spikelet and glume morphology (de Wet et al., 1983c). Consequently, the common names Japanese and Indian barnyard millet have been suggested to simplify research and investigation of their phylogeny (Yabuno, 1987). Despite having such strong phenotypic similarities, cytology and marker work have shown the two millets to be genetically distinct; F_1_ hybrids of the two species are sterile (Yabuno, 1962; Hilu, 1994). Both species are known for their fast maturity, high storability, and the ability to grow on poor soil (Yabuno, 1987). ICRISAT currently holds 743 accessions of these barnyard millets from nine countries, with a core collection of 89 varieties recently established (Upadhyaya et al., 2014). Other significant collections can be found at NIAS and the USDA (Hilu, 1994). Sequence data and genetic map availability for both millets are generally low (Dwivedi et al., 2012). Initial transgenic work has been reported on the Japanese variety, but callus regeneration protocols have been reported for both species (Gupta et al., 2001; Kothari et al., 2005).

In addition to the two cultivated species, research has also been conducted on 20–30 wild *Echinochloa* barnyard millet relatives, some of which have agriculturally interesting traits including rice-mimicry and perennial growth habit. Hybridization within the genus is rampant, and is thought to have contributed to the evolution and current diversity of barnyard millets (Hilu, 1994; Yamaguchi et al., 2005).

#### Japanese barnyard millet (*Echinochloa esculenta*)

Japanese barnyard millet originated in eastern Asia (Figure 2) from its wild counterpart *E. crus-galli*, “barnyard grass” (Yabuno, 1987; Hilu, 1994). It can be differentiated from the Indian species by its larger, awned spikelets with glumes that appear papery instead of membranous (de Wet et al., 1983c). It is tolerant to cold and was historically grown in areas where the climate or land did not suit rice production, particularly in the north of Japan (Yabuno, 1987). In Japan, folklore states that barnyard millet originated from the dead body of a god. Along with proso millet, it makes up part of the “Gokoku,” a general term for five staple grains (Yabuno, 1987). Japanese barnyard millet has been found in the coffins of 800-year-old mummies from the Iwate prefecture, and documents from the 1700s list different cultivars organized by maturity time (Yabuno, 1987). Its historical importance might be attributed to the relief it provided in times of rice crop failure. However, Japanese barnyard millet production has sharply decreased in the last century due to the introduction of cold-tolerant rice varieties and better irrigation practices (Yabuno, 1987). Nevertheless, today it remains the most common millet consumed in Japan, with reported health benefits common to many of the small millets such as its ability to lower plasma glucose concentration, insulin, adiponectin and tumor necrosis factor-α when fed to diabetic mice (Nishizawa et al., 2009). The protein content of Japanese barnyard millet is twice as high as that of rice (Yabuno, 1987). Across genotypes there is diversity in the levels of proteins and healthy lipids, with one genotype suggested as having particularly beneficial antioxidant activity (Kim et al., 2011).

Unlike other small millets consumed in East Asian countries such as foxtail and proso, barnyard millet has no glutinous variety. However, some landraces have been identified which contain very low levels of amylose due to a deletion in one of three *waxy* genes. One such landrace, “Noge-Hie,” was treated with γ-radiation resulting in progeny lacking the Waxy (Wx) protein (Hoshino et al., 2010). The trait was stably inherited, and this new glutinous variety (“Chojuromochi” in Japan) might be useful for increasing demand for millet products among Japanese consumers.

The morphological and physiological diversity of Japanese barnyard millet is suggested to be high (Nozawa et al., 2006). Flowering time, inflorescence shape, and spikelet pigmentation, among other features, vary across landraces. The species can be grouped into the races *utilis* and *intermedia* (Upadhyaya et al., 2014). Molecular diversity studies for Japanese barnyard millet have begun using the non-coding regions of chloroplast DNA as well as nuclear molecular markers (RAPDs, SSRs) and isozymes, although these studies appear to be limited in their sample number (Hilu, 1994; Nakayama et al., 1999; Yamaguchi et al., 2005; Nozawa et al., 2006). Though DNA sequence information in Japanese barnyard millet is otherwise lacking, studies performed on the closely related barnyard grass (*E. crus-galli)* have generated important sequence information. For example, extensive transcriptomic profiling and annotation have been performed on herbicide resistant varieties of barnyard grass resulting in 74 ESTs, which might be adapted to the study of the cultivated relative (Li et al., 2013; Yang et al., 2013).

#### Indian barnyard millet (*E. frumentacea*)

Indian barnyard millet, or sawa, was domesticated in India (Figure 2) across its current range from its wild counterpart *E. colona*, “jungle rice” (Yabuno, 1987; Hilu, 1994). In India, this millet is either harvested as a weed along with a main crop or is grown in a mixture with finger millet and foxtail millet (Gupta et al., 2009b). It is generally cultivated on hilly slopes in tribal areas where few other agricultural options exist and is indispensible in the northwest Himalayan region (Gupta et al., 2009b). Quick maturity makes the species well-adapted to regions with little rainfall (Channappagoudar et al., 2008). Indian barnyard millet contains antifeedants which are present at concentrations higher than in rice, and it displays resistance to the feeding activity of brown planthopper (Kim et al., 2008). In central Africa it is fermented to make beer or used for food, and has been found in the intestines of pre-dynastic Egyptian mummies (de Wet et al., 1983c). When fed to diabetic humans, significant reductions of blood glucose levels and LDL cholesterol have been reported (Ugare et al., 2014).

Significant phenotypic variation is observed in Indian barnyard millet. Four morphological races (*laxa, robusta, intermedia*, and *stolonifera*) were recognized by de Wet in 1983 based on the lengths of flag leaves, peduncles, inflorescences, racemes, as well as plant height and basal tiller number. Race *laxa* is endemic to the Sikkim Himalayas and only available in a few collections (de Wet et al., 1983c). More recently, a variety of morphological parameters were examined, and principle component analysis (PCA) indicated three morphotypes corresponding to races *robusta, intermedia*, and *stolonifera; laxa* was absent suggesting that efforts must be made to collect more of this race (Gupta et al., 2009b). The authors saw high variability in grain yield, straw yield, and number of productive tillers. They report that the number of racemes, flag leaf width, and internode length showed high correlation with grain yield and should be considered by breeders when performing selections, and promising donor genotypes of these and other traits have been reported (Channappagoudar et al., 2008; Gupta et al., 2009b). Variation across genotypes in photosynthesis and related traits such as transpiration and stomatal conductance has also been observed (Subrahmanyam and Rathore, 1999). Grain smut (*Ustilago panici-frumentacei*) is a major hindrance of yield, but progress has been made in advanced breeding lines which display low susceptibility when compared to other accessions in which high variability remains (Gupta et al., 2009a).

An early study (Hilu, 1994) using RAPD markers suggested that the sequence diversity of Indian barnyard millet is significantly higher than the Japanese species, perhaps because of multiple domestication events in different locations across India (Hilu, 1994). Variation of markers was 44%, which is high when considering the inbreeding nature of the crop (Hilu, 1994). However, more comprehensive studies are needed that utilize a greater number of molecular markers and genotypes. Similarly, DNA sequence analyses are lacking in Indian barnyard millet.

### Little millet (*Panicum sumatrense*)

Also called *sama*, little millet is cultivated to a limited extent in India, Sri Lanka, Pakistan, Myanmar, and other South East Asian countries (Table 1) (Hiremath et al., 1990). In India it is important to tribes of the Eastern Ghat mountains and grown in combination with other millets (Hiremath et al., 1990). Little millet is a domesticated form of the weedy species *Panicum psilopodium* (de Wet et al., 1983a). The chromosomes of hybrids of *Panicum sumatrense* and *P. psilopodium* pair almost perfectly with only a single quadrivalent, indicating that divergence between the two species may have initially occurred through a single reciprocal translocation (Hiremath et al., 1990). Hybrid plants are fertile and vigorous with non-shattering spikelets, and thus introgression of genes between the two species is common (Hiremath et al., 1990). This hybridization ability combined with its wide range of cultivation across India suggests that little millet was domesticated independently several times, although exact dates remain undetermined (de Wet et al., 1983a). Little millet is comparable to other cereals in terms of fiber, fat, carbohydrates, and protein, and rich in phytochemicals including phenolic acids, flavonoids, tannins, and phytate (Pradeep and Guha, 2011). Like many other small millets, it is drought, pest and salt tolerant (Sivakumar et al., 2006b; Bhaskaran and Panneerselvam, 2013; Ajithkumar and Panneerselvam, 2014). The time to maturity for most cultivars is about 90 days (de Wet et al., 1983a).

Little millet is divided into two races based on panicle morphology, *nana* and *robusta*. Race *nana* matures faster and produces less biomass than *robusta* (de Wet et al., 1983a). In a tribal area of the Indian Kolli hills, diversity among locally grown landraces of little millet was found to be high for all morphological traits measured both within and between landraces despite a small sampling area (Arunachalam et al., 2005). High diversity, heritability and genetic advancement was observed in terms of yield and productive tillers in a collection of 109 landraces, meaning that the crop might be a good candidate for varietal development (Nirmalakumari et al., 2010). A different collection of 460 accessions of little millet held by ICRISAT displayed genetic variation for most of the traits examined (Upadhyaya et al., 2014). A core collection of 56 genotypes was identified which was representative of the entire seed bank. Increased heritable lodging resistance has been introduced to a population of little millet with γ-ray mutational breeding (Nirmalakumari et al., 2007).

The molecular biology of little millet has been explored to a limited extent. As part of a study to identify seven millet species based on their chloroplast DNA, the *trnS-psbC* gene region was characterized and subjected to RFLP analysis (Parani et al., 2001). This study showed that it was possible to distinguish all the millet species when the enzymes *Hae*III and *Msp*I were used in combination. To investigate mechanisms behind little millet's high prolamine content, a zein-like storage protein was isolated and sequenced (Sivakumar et al., 2006a). Furthermore, α-amylase from little millet has been isolated and characterized in terms of biomass and optimum pH (Usha et al., 2011). To the best of our knowledge, no protocols for callus regeneration or transgenic technology have been published. Little millet is perhaps the least studied of the small millet species and there is much that requires investigation, including the establishment of a genetic map and sequenced genome.

## Trends, gaps and recommendations on how to foster diversity within orphaned small millets for the new green revolution

The World Summit on Food Security has set a target of 70% more food production by 2050, requiring annual increases of 44 million tons, 38% above current annual increases (Tester and Langridge, 2010). Climate change will cause additional difficulties as many regions are becoming drier with increasingly severe weather patterns (Dai, 2011), and fossil-fuel based nitrogen use is increasingly restricted by legislation intended to slow climate change (Tester and Langridge, 2010). The small millets have the potential to meet these challenges, given their drought tolerance and ability to grow under low input conditions, along with other health-promoting traits valued by humans. Unfortunately, the small millets suffer from low yields (only 0.8 tons grain per hectare) (Plaza-Wüthrich and Tadele, 2012). For the small millets to succeed, priority traits for breeding will need to include improving yield under stress conditions (low input, salt, drought, pests, pathogens). Fortunately, an attractive feature of the small millets is that they continue to be cultivated in remote areas which has preserved their biodiversity, giving breeders potential access to unique genes for crop improvement. Due to limited resources, however, current efforts thus far have concentrated primarily on characterizing and reporting the extensive diversity present in seed banks, with few genetic and genomic tools available to exploit this biodiversity for crop improvement. A further challenge in some species (e.g., foxtail millet) is persistent cross-hybridization with wild relatives. Improved varieties of small millets could play a role in the “New Green Revolution”—a term coined to reflect novel strategies which will be required to deal with complex challenges in developing nations including increasing population and ever-diminishing arable land (Den Herder et al., 2010).

### Exploiting diversity within seed banks

Diversity is the basis of crop improvement. As described in this review, the small millets possess considerable morphological and genetic sequence variation that can be used by breeders to generate improved varieties. Seed banks across the globe conserve collections of small millets as shown in Table 2, but a challenge is that less diverse germplasm is available for species that are cultivated in a limited geographic region. For example, little millet, which is mainly grown in the Eastern Ghats of India, is represented by a collection of only 466 accessions (Upadhyaya et al., 2014). By contrast, ICRISAT currently holds 6804 accessions of finger millet, a crop widely grown on 1.8 million ha throughout India with extensive cultivation in Eurasia and Africa^3^. Core collections follow the same patterns, with several reported for finger millet but only one for little millet (Upadhyaya et al., 2014). It is essential that core collections be established for all of the millets, however, especially at larger seed banks, to facilitate efficient trait selection. As modern small millet cultivation for human consumption typically occurs in poor nations (with some exceptions), the seed bank infrastructure and associated reporting in the scientific literature and in online databases is sparse and difficult for breeders from foreign nations to access. Furthermore, trait descriptions for each accession are often not reported. Improved funding, coordination, communication and sharing of genetic resources are needed to overcome these problems.

### Harvesting genes from the wild

Though interspecific hybrids between some cultivated and wild millets can be problematic, the wild relatives of the small millets may serve as donors of useful genes for crop improvement (e.g., herbicide resistance). To enable breeding, the hybridization ability of Indian and Japanese barnyard millet (Yabuno, 1962; de Wet et al., 1983c) may thus serve as an advantage. However, full realization of this breeding potential may require embryo rescue techniques to bring weak F_1_ progeny to adulthood (Plaza-Wüthrich and Tadele, 2012) and better access by breeding programs to wild germplasm (Hajjar and Hodgkin, 2007). Today, the wild germplasm is sometimes studied only from a weed science perspective (Peterson and Nalewaja, 1992; Dilday et al., 2001).

### Combining traditional knowledge of diversity with modern techniques

Small millets are often grown in remote regions of the world, and hence significant traditional knowledge of millet diversity persists that can serve as a valuable resource for crop improvement. Isolated farming communities often cultivate dozens of locally known millet landraces that are valued for a wide variety of traits (e.g., short duration to combat delayed rains as the result of climate change). Farmers use a complex system to classify their landraces, and in some instances this classification is considered more informative than scientific phylogeny (Rengalakshmi, 2005). On the opposite end of the technological spectrum, research using simple DNA barcoding *in lieu* of larger numbers of molecular markers is being attempted to classify the small millets down to the landrace level (Newmaster et al., 2013). A unique opportunity in the small millets is combining traditional knowledge with molecular techniques to characterize diversity for the purposes of crop improvement.

### The need for complete linkage maps, molecular markers and genome sequences

As described above, in some species, markers including RFLPs, AFLPs, ESTs, and SSRs have been linked to beneficial traits including stress tolerance (Lata et al., 2011). Other, less conventional selective biomarkers have been suggested including differing ratios of transcription factors under stress (Gupta et al., 2014a). However, several small millets lack molecular and genetic markers (e.g., little millet and kodo millet) and no robust linkage maps appear to exist (Dwivedi et al., 2012). Genome and EST sequencing efforts will assist in the development of molecular markers in these species, along with using reference genomes (e.g., from major cereal relatives) to identify orthologous markers. Currently, only the foxtail millet genome has been sequenced and published (Bennetzen et al., 2012; Zhang et al., 2012).

### Advances in transgene research and molecular mechanisms

As noted in this review, detailed protocols for callus regeneration and transgene protocols have been published for all small millet species except little millet (Kothari et al., 2005; Ceasar and Ignacimuthu, 2009; Plaza-Wüthrich and Tadele, 2012). Since small millet women farmers toil in the drudgery of removing weeds manually (Rengalakshmi, 2005), an attractive transgene trait may be glyphosate herbicide resistance (RoundupReady).

As the small millets are respected by traditional farmers for their extreme abiotic and biotic stress resistance, an understanding of the molecular mechanisms underlying these traits may lead to agronomic improvement of related major cereals. Unfortunately millet diversity remains largely unexplored at the level of molecular mechanism, with the exception of a limited number of studies noted earlier. One especially attractive target will be to understand the ability of barnyard millet to grow under extremely low nitrogen conditions.

### Socio-economic constraints

**Figure 3:**
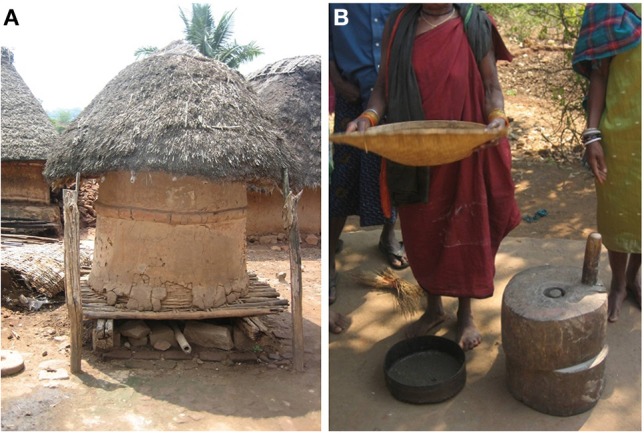
**Indigenous technologies and practices of modern small millet farmers**. **(A)** A typical granary in the Eastern Ghats of India used for small millet storage. **(B)** A woman farmer in Northern India holds a basket used for separating millet grain from chaff. She stands beside a manual millstone used for grinding millet grain into flour. Source: M. Raizada.

Despite the promise of the small millets, various socio-economic constraints have limited their consumption and hence contributed to a loss of cultivated diversity:

First, a major reason why the small millets are declining in production is that these crops are typically labor-intensive; women are often responsible for manual post-harvest processing, grain threshing and milling (Rengalakshmi, 2005) (Figure 3). To overcome this obstacle, inexpensive machinery is needed.

As noted above, a second challenge to greater adoption of small millets is their comparatively low yield (Plaza-Wüthrich and Tadele, 2012) as a result of the lack of scientific attention. However, the benefits of adding millet to the cropping system may outweigh the drawbacks of low yield (e.g., to combat local protein deficiency or crop failure in stressful environments) (Plaza-Wüthrich and Tadele, 2012). Furthermore, the small millets can be grown in very stressful environments, where major cereals may fail.

Third, family-farm-level diversity is heavily affected by community access to seed which may be limited by current rural seed systems (Nagarajan et al., 2007). However, the presence of local seed markets has been found to increase millet diversity indicating that such markets may serve as good points of introduction for improved varieties.

Finally, agricultural policies in different nations have negatively impacted the cultivation and research of small millets. Production in many areas is becoming displaced by mainstream cereals: in Kenya, the focus has been placed on the cultivation of maize instead of finger millet (Dida et al., 2008), while in Northern Japan, cold-tolerant rice has almost completely replaced barnyard millet (Yabuno, 1987). Reduced cultivation of these millets in financially-rich countries like Japan is problematic, because it may decrease global research funding for these crops. However, recent reports revealing medicinal and nutritional benefits of these species (absence of gluten, cancer inhibition, control of blood-glucose and cholesterol) might catalyze consumer interest and hence funding in the developed world (Hegde et al., 2005; Nishizawa et al., 2009; Kim et al., 2011; Zhang et al., 2014). Nevertheless, landraces from these areas should be preserved in seed banks to ensure their conservation.

Given these socio-economic constraints, millets must not be blindly advocated in the developing world in biodiversity strategies. Prior to their introduction, multi-disciplinary surveys must be undertaken with local farmers concerning their nutrition, seed availability, economy, climate, and other crops in the cropping system.

## Conclusions

Modern agriculture is characterized by dominance of a few crop species with a trend toward genetic homogenization as a result of the global exchange of alleles via breeding. In contrast, traditional farmer landraces of the small millets continue to be cultivated under relative genetic isolation, and hence provide living examples of genetic and phenotypic biodiversity in contemporary agriculture. The small millets are valued by traditional farmers for their nutritional content and health promoting properties, ability to grow under low input conditions and tolerance to extreme environmental stress, especially drought. In a world facing limiting natural resources and climate change, these crops thus hold tremendous potential as valuable instruments in the toolkit of the New Green Revolution. It is hoped that germplasm resources combined with modern genomic tools can help to accelerate exploitation of this biodiversity.

## Author contributions

Both TG and MR conceived of the manuscript. TG wrote the manuscript and MR edited the manuscript. Both authors read and approved the final manuscript.

### Conflict of interest statement

The authors declare that the research was conducted in the absence of any commercial or financial relationships that could be construed as a potential conflict of interest.

## Footnotes

^1^http://bioinnovate-africa.org/about-us/news/item/162-finger-millet-genomics-project-to-provide-researchers-with-better-tools-for-variety-production

^2^http://www.ars-grin.gov/

^3^http://www.icrisat.org/crop-fingermillet.htm

## Abbreviations

EST: expressed-sequence tag
RFLP: restriction fragment length polymorphism
AFLP: amplified fragment length polymorphism
SSR: simple sequence repeat
WUE: water use efficiency
NUE: nitrogen use efficiency.
